# Malrotation and Midgut Volvulus associated with Asymptomatic Duplication Cyst of Jejunum

**DOI:** 10.21699/ajcr.v7i4.447

**Published:** 2016-09-01

**Authors:** Sandip Kumar Rahul, Vijai Datta Upadhyaya, Basant Kumar

**Affiliations:** Department of Pediatric Surgery, Sanjay Gandhi Post Graduate Institute of Medical Sciences, Lucknow, India

**Keywords:** Gastrointestinal duplication, Malrotation, Midgut volvulus

## Abstract

Gastrointestinal duplications can affect any part of the alimentary tract and are notorious for their variable presentation. Their association with malrotation and midgut volvulus is rare. We describe an 8-year old boy presented with episodes of abdominal pain. Radiological workup showed whirlpool sign and abnormal relationship of mesenteric vessels. At operation, malrotation with chronic volvulus was found. Incidentally, a jejunal communicating duplication cyst was also noted.

## CASE REPORT

An 8-year-old boy presented with on and off abdominal pain for four months which increased in frequency and intensity 24 hours before presentation. Pain used to worsen on oral intake and was occasionally associated with non-bilious vomiting. There were no other significant symptoms. He had been evaluated elsewhere with a normally reported contrast follow-through study done three months earlier.

On examination patient weighed 20 kg and was dehydrated. There was no pallor and the patient was afebrile. Abdomen was soft, non-distended and non-tender without any palpable mass. Bowel sounds were increased. Per rectal examination was normal. After admission, he had episodes of agonizing abdominal pain. Nasogastric tube revealed non bilious aspirate. Routine blood investigations were within normal limits with baseline hemoglobin of 10.4 g/dl. Abdominal x-ray did not reveal any pathology. Abdominal ultrasound (USG) showed positive whirlpool sign. CT scan of the abdomen confirmed the USG findings and showed the abnormal relationship of superior mesenteric vein and artery (Fig.1). Considering the acute symptoms and the radiological findings, patient was taken up for emergency surgery. At laparotomy, midgut volvulus with malrotation was found. Derotation of the twisted small bowel loops was done. Bowel loops were healthy and viable. Thick Ladd’s bands with narrow small bowel mesentery were present. Ladd’s bands were divided and mesentery widened. Approximately 40 cm distal to the duodeno-jejunal flexure, a 10cm x 5cm tubular jejunal duplication on the mesenteric side was found (Fig. 2). It communicated with the normal jejunum through a narrow opening. Resection of the involved segment with end to end jejuno-jejunal anastomosis was performed. Resected specimen was sent for histopathological examination. Postoperative period was uneventful. Patient has been asymptomatic in the follow up period. Histopathological examination showed normal small intestinal features with ectopic gastric mucosa.

**Figure:**
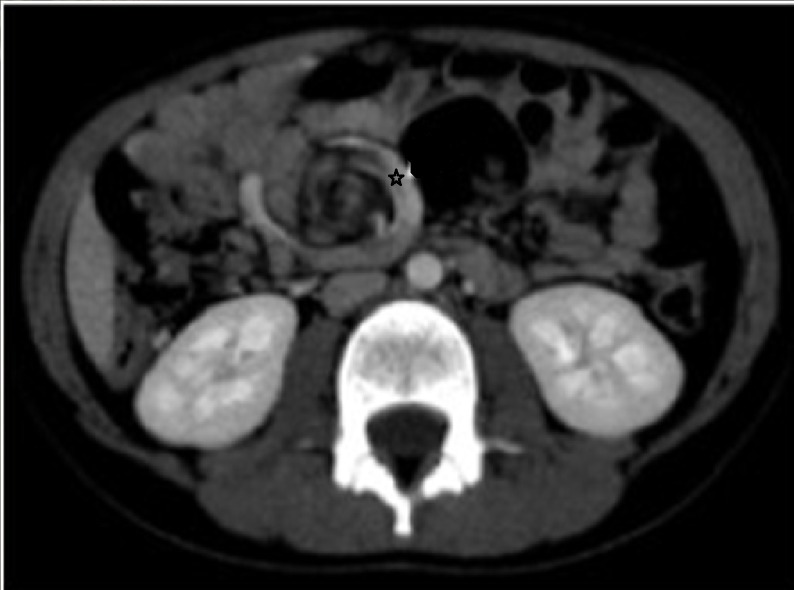
Figure 1: Twisted mesenteric vessels seen on CECT scan (Whirlpool sign).

**Figure:**
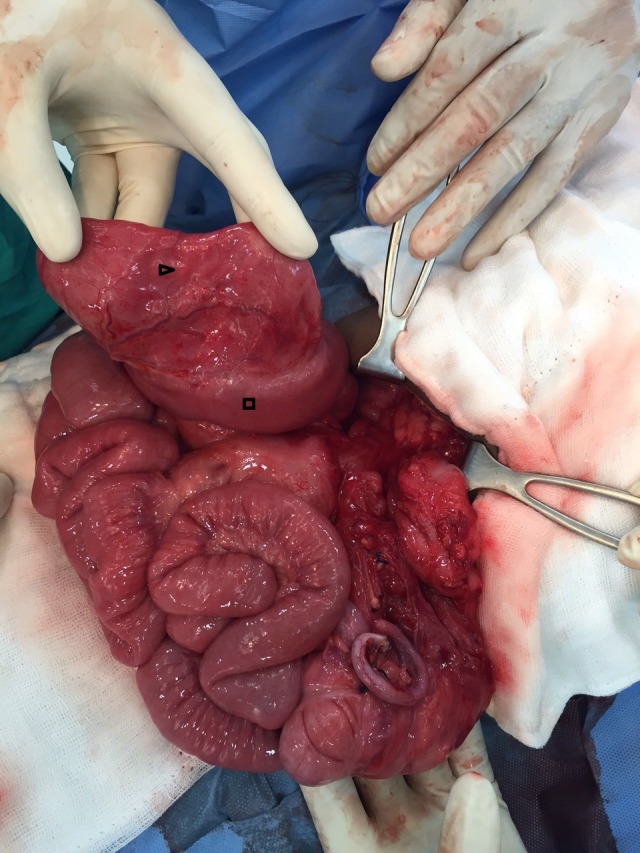
Figure 2: Tubular Jejunal duplication (∆) on the mesenteric side of proximal jejunum (□). Mobile cecum can be appreciated along with congestion of small bowel owing to chronic volvulus.

## DISCUSSION

Although both malrotation and gastrointestinal duplications are important causes of intestinal obstruction in the paediatric age group, finding them in the same patient has been described to be rare in literature. [1- 4] We could not find the exact incidence of this association.

Despite having both malrotation and jejunal duplication, our patient seldom experienced any symptom before four months of presentation to the hospital. Chronic malnutrition leading to low weight for age and repeated episodes of pain abdomen can both be explained by chronic volvulus and malrotation.[5] Even when not causing frank Intestinal obstruction, malrotation has been found to delay gastric emptying and aggravate gastro-esophageal reflux.[6] This mechanism can explain non-bilious vomiting and aspirates in nasogastric tube. Jejunal duplication could also have added to the pain and relative intestinal obstruction. No pallor or history of melena signifies that the patient did not have bleeding despite the presence of ectopic gastric mucosa. It is possible that during episodes of volvulus the involved midgut emptied to some extent into the communicating duplication and derotated after becoming less bulky. Possibly, this mechanism of relief of obstruction may be the cause for the child to be asymptomatic till this age and also for the non-bilious vomiting and aspirate. Unfortunately, the patient did not have images of the contrast study done outside and it could not be repeated due to his acute symptoms otherwise a communicating duplication could be picked preoperatively. Finding of whirlpool sign on imaging and abnormal relationship of mesenteric vessels led us to preoperative diagnosis of malrotation and volvulus.

We concluded that malrotation and jejunal duplication have rare association and USG coupled with a carefully done contrast study is indispensable in such cases to delineate the abnormal anatomy preoperatively.

## Footnotes

**Source of Support:** Nil

**Conflict of Interest:** None declared

